# Primary Myelofibrosis Occurring during Targeted Therapy for Chronic Lymphocytic Leukemia: A Report of Two Cases

**DOI:** 10.3390/curroncol29030122

**Published:** 2022-02-27

**Authors:** Francesco Angotzi, Andrea Visentin, Federico Scarmozzino, Alessandro Cellini, Roberta Bertorelle, Marco Pizzi, Gianni Binotto, Angelo Paolo Dei Tos, Livio Trentin

**Affiliations:** 1 Hematology and Clinical Immunology Unit, Department of Medicine, University of Padua, 35128 Padova, Italy; francesco.angotzi@studenti.unipd.it (F.A.); andrea.visentin@aopd.veneto.it (A.V.); alessandro.cellini@studenti.unipd.it (A.C.); gianni.binotto@aopd.veneto.it (G.B.); 2 Surgical Pathology and Cytopathology Units, Department of Medicine, University of Padova, 35100 Padova, Italy; federico.scarmozzino@studenti.unipd.it (F.S.); marco.pizzi@aopd.veneto.it (M.P.); angelopaolo.deitos@aopd.veneto.it (A.P.D.T.); 3 Immunology and Molecular Oncology Unit, Veneto Institute of Oncology, IOV-IRCCS, 35128 Padova, Italy; roberta.bertorelle@iov.veneto.it

**Keywords:** chronic lymphocytic leukemia, primary myelofibrosis, ibrutinib, venetoclax

## Abstract

The disease course of chronic lymphocytic leukemia (CLL) is frequently characterized by the occurrence of various complications, such as second primary cancer, which can impact patients’ prognoses. While therapies for CLL have evolved tremendously in the past decades, overlooking the possibility of rare neoplasms that arise along with CLL may hinder the benefit that these therapies grant to patients. Moreover, the ability of newer therapies to alter the landscape of these complications is still largely unknown. Primary myelofibrosis (PMF) is not commonly associated with CLL, with only a few cases reported in the literature, with little information regarding the clinico-biological features and the optimal management for these associated conditions. Here, we report two unusual cases of PMF that occurred a few months after the start of therapy for CLL with targeted agents (ibrutinib and venetoclax). Both cases represented a diagnostic and therapeutic challenge, underscoring the need for clinicians to remain vigilant about the possible co-occurrence of these two hematological malignancies, especially in the era of targeted therapy for CLL.

## 1. Introduction

Chronic lymphocytic leukemia (CLL) is an indolent mature B-cell neoplasm accounting for the majority of adult leukemias in the western world. In the last few years, the treatment of CLL has greatly changed, due to the introduction of targeted agents that significantly improve the prognosis of patients with adverse prognostic features [1]. Patients affected by CLL develop several complications that have an impact on survival, including infections, autoimmune diseases and second primary cancers (SPC) [2,3,4]. The latter include melanoma, non-melanoma skin cancers and non-Hodgkin lymphomas [4]. Myeloproliferative neoplasms (MPN), such as polycythemia vera, essential thrombocytosis (ET) and primary myelofibrosis (PMF), rarely occur in CLL patients, with only 28 cases of concurrent CLL and PMF reported in the literature so far. Among these cases, the diagnosis of PMF is frequently reported several years after CLL, without any clear-cut association between the type of administered therapy and PMF development, but with growing evidence that these two diseases may be more intertwined than previously thought [5,6,7]. None of those cases were diagnosed while patients were on targeted therapies with either ibrutinib or venetoclax.

Here, we report two cases who developed PMF in the context of CLL during targeted therapy with ibrutinib and venetoclax, respectively.

## 2. Case Reports

**Figure 1:**
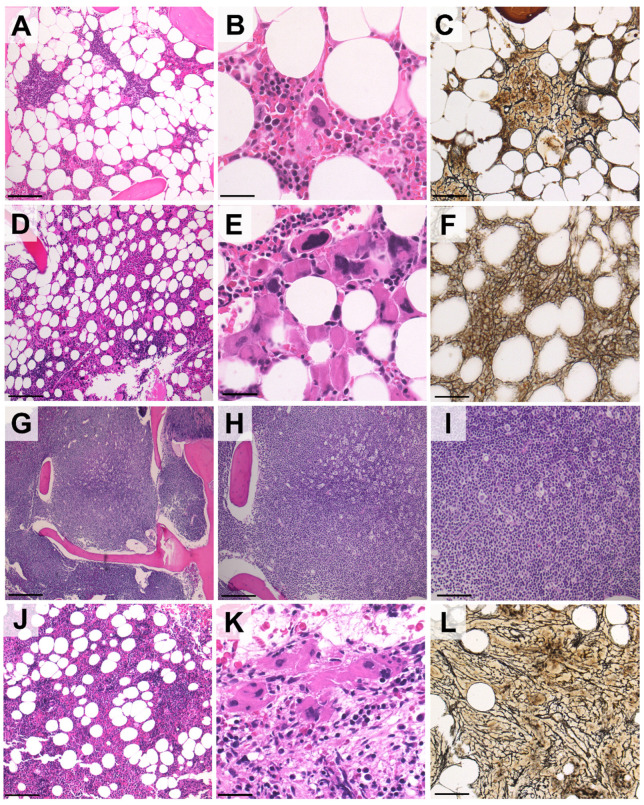
Representative histological features of post-CLL PMF following treatment with ibrutinib or venetoclax. (**A**–**C**). In patient #1, the BM before ibrutinib therapy was normocellular (**A**) with maturing hematopoietic cells and a nodular infiltrate of small lymphocytes consistent with CLL (**B**). Mild reticulin fibrosis was associated with CLL nodules (**C**). (**D**–**F**) Six months into ibrutinib therapy, BM cellularity was increased (**D**) and large, tight clusters of atypical megakaryocytes with bulbous nuclei were present (**E**). A reticulin stain disclosed diffuse MF-2 grade fibrosis, prompting the diagnosis of PMF (fibrotic stage) (**F**). (**G**–**I**). Areas of bone marrow in patient #2 before venetoclax therapy, showing diffuse infiltration by CLL cells. (**J**–**L**) Nine months after venetoclax was started, the bone marrow of patient #2 also presented tight clusters of megakaryocytes and MF-2 grade fibrosis ((**H**,**E**) and reticulin stains; original magnification: 10×, 20× and 40×; Scale bar (**A**,**C**,**D**,**F**,**I**,**L**): 100 µm; (**B**,**E**,**K**): 50 µm; (**G**,**H**,**J**): 200 µm).

**Figure 2:**
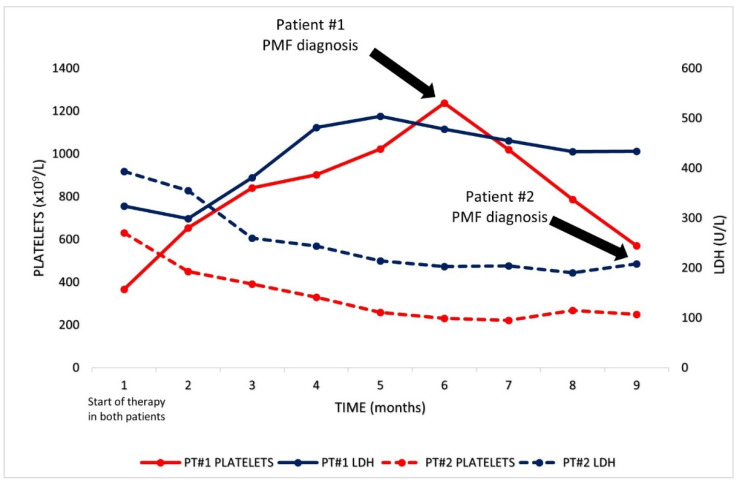
Graphical representation of platelets and LDH levels of both patients from the start of therapy with either ibrutinib or venetoclax until PMF diagnosis.

An 82-year-old male, diagnosed with CLL in 2013, was referred to our institution with progressive disease in 2017. The patient’s only complaint was generalized pruritus, with a maculo-papular rash deemed secondary to CLL. The full blood count showed lymphocytosis, with the presence of atypical lymphocytes on the blood smear (Hb 149 g/L, PLT 340 × 10^9^/L, WBC 37 × 10^9^/L, PMN 11 × 10^9^/L, and lymphocytes 26 × 10^9^/L). A flow cytometric analysis on the peripheral blood (PB) documented a B-cell clone expressing CD5, CD23, CD20 (dim), CD38 and CD200, consistent with CLL; cytogenetics and molecular studies documented chromosome 11q deletion, with an unmutated IGHV status, and wild-type *NOTCH1* and *TP53* genes. A whole-body CT scan reported multiple enlarged cervical, axillary, mediastinal and abdominal lymph nodes (maximum diameter: 48 mm), with concurrent splenomegaly (16 cm). A bone marrow (BM) biopsy showed diffuse infiltration by CLL cells. Treatment with obinotuzumab plus chlorambucil, for six cycles, was started in October 2017, achieving a partial response (PR) 8 months later. A post-therapy BM biopsy disclosed normal cellularity with trilinear maturing hematopoiesis, minimal CLL infiltration (10% of total cellularity) and mildly increased reticulin fibrosis (MF-1) (Figure 1A–C). In November 2019, the disease relapsed, with multiple enlarged lymph nodes in the abdomen and splenomegaly documented by CT scan, so therapy with ibrutinib (420 mg/day) was started. Of note, the platelet counts had been mildly elevated since 2018, with a peak value of 638 × 10^9^/L. Since the start of ibrutinib therapy in November 2019, however, thrombocytosis progressively worsened, with platelet counts up to 1.2 × 10^12^/L at 6 months into ibrutinib therapy, with no associated symptoms besides a continuous headache (Figure 2, continuous lines). During the same time, the lactic dehydrogenase (LDH) levels also steadily increased from 324 to 504 U/L (Figure 2). These findings prompted another bone marrow examination, which now showed hypercellular trilinear hematopoiesis with tight clusters of atypical megakaryopoiesis, MF-2 grade fibrosis and moderate CLL infiltration (15–20% of the overall cellularity) (Figure 1D–F). Molecular tests on PB documented the *JAK2-V617F* mutation with an allele burden of 4.7%, supporting the diagnosis of primary myelofibrosis. To assess the presence of the mutation on CLL cells, the same analysis was performed on isolated lymphocytes, using PCR, showing a negative result. Thus, treatment with hydroxyurea (1000 mg/day) was started alongside ibrutinib, and, 8 months later (January 2021), gradual normalization of the platelet counts was observed (Hb 109 g/L, PLT 220 × 10^9^/L, WBC 4.45 × 10^9^/L with 72% lymphocytes). At present, the patient remains asymptomatic, with both diseases being controlled by dual ibrutinib and hydroxyurea therapy.

A 74-year-old man with a history of CLL since 2005, who was previously treated with fludarabine, cyclophosphamide and rituximab (FCR), presented with disease progression in May 2020. The disease disclosed adverse cytogenetic and molecular features (13q and 17p deletions; mutated *TP53*, an unmutated IGHV status and wild-type *NOTCH1*). At this time, the patient was asymptomatic, and the blood counts were remarkable for mild anemia and lymphocytosis with a normal platelet count (Hb 119 g/L, PLT 283 × 10^9^/L, WBC 56.03 × 10^9^/L with 79% lymphocytes). Elevated LDH levels were also noted (393 U/L). A CT scan was performed, which documented multiple lymphadenopathies on both sides of the diaphragm (maximum diameter: 52 mm) and an enlarged spleen (diameter: 22 mm). A BM biopsy showed a diffuse infiltrate of CLL cells (Figure 1 G–I). Splenomegaly was already documented in 2015, but, by this time, the spleen had grown by an additional 4 cm. According to prognostic factors and the patient’s preferences, fixed-duration therapy with rituximab plus venetoclax for 24 months was started in June 2020, with progressive venetoclax dose escalation up to 400 mg/day. The therapy was well tolerated and led to progressive lymphocyte count normalization over 2 months (from 44.46 × 10^9^/L to 4.02 × 10^9^/L). A follow-up CT scan, performed 8 months after the start of therapy, documented a PR with regression of most lymphadenopathies, but persistent splenomegaly, without any variation in spleen size. Moreover, progressive mild thrombocytopenia developed immediately following the start of therapy and persisted in the following months, reaching counts of 101 × 10^9^/L after 8 months (Figure 2, dotted lines). Given these findings, a BM biopsy was performed, which showed hypercellular hematopoiesis (75% total cellularity), with increased numbers of atypical megakaryocytes, MF-2 fibrosis and mild CLL infiltration (10% of BM cells) (Figure 1K–L). PCR and DNA sequencing on PB led to the identification of the driver, the MPL-*W515K* mutation, confirming the diagnosis of PMF. Again, the results from an analysis of the mutation by PCR on isolated CLL cells were negative. Given the near-normal blood counts and the absence of symptoms related to PMF, no additional treatment, directed at PMF, was administered. At present, the patient is alive and in a good clinical condition on venetoclax therapy, which will be discontinued in July 2022.

The cases reported here document the unusual association between CLL and PMF upon treatment with ibrutinib and venetoclax. In both of our patients, CLL preceded PMF diagnosis by many years (8 and 18, respectively). The median time between the diagnosis of a lymphoproliferative disorder and an MPN was previously reported to be 42.6 months, which is significantly shorter than the time observed in our patients [6]. PMF was hardly suspected during the course of the disease, since neither patient presented with typical MPN-related signs or symptoms. In the first case, the unexpected development of severe thrombocytosis was clinically puzzling and prompted BM evaluation for an underlying MPN. Although mild thrombocytosis had been present since the first BM evaluation, at that time, PMF was excluded by the seemingly normal hematopoiesis. Notably, in this case, a mild degree of reticulin fibrosis cannot be assumed as a hint to an underlying PMF, as BM fibrosis is a known pathological finding associated with CLL, especially in cases with chromosome 11q deletion [8]. Indeed, both cases showed morphological characteristics that are peculiar to PMF, which are never found in secondary fibrosis, such as tightly packed megakaryocytes, with varying degrees of dysplasia. Even in the second patient, the development of PMF was unexpected, and the correct diagnosis was prompted by the persistence of splenomegaly after treatment. However, it cannot be excluded that some signs of PMF or prefibrotic PMF existed under the sheet of CLL cells.

While rare, the association of CLL with PMF and other MPNs may not be incidental, and could disclose recurrent clinical and molecular features, which are in keeping with our cases. A recent review of the literature by Darawshy et al., which collected data from other case reports and retrospective series, has, indeed, found that PMF is frequently diagnosed several years after CLL, and suggested the role of the JAK2-V617F driver mutation and t(1;6) as the main genetic abnormalities [7]. While both of our patients harbored either a JAK2 or MPL mutation, which is commonly associated with PMF, these may not be the sole determinants of PMF development in CLL. Indeed, other recurrent somatic mutations have been found in both MPN and CLL, which might create a common genetical milieu that favours the occurrence of both diseases [9]. Moreover, the JAK2 mutation in CLL has been identified up to nine years prior to PMF diagnosis [9]. This is particularly in keeping with our first case, who presented with thrombocytosis years before PMF diagnosis, thus suggesting the possible pre-existence of an MPN, which only became clinically overt after therapy with ibrutinib was started. In addition, the fact that, in both our patients, the JAK2 and MPL mutations were not found on isolated CLL cells supports the hypothesis that the concurrent manifestation of CLL and PMF might originate from the proliferation of two neoplastic clones under a common genetic background and oncogenic stimulus.

The prognostic impact of PMF on CLL patients is unknown, as most reported cases only provide limited follow-up data. The occurrence of PMF is intuitively detrimental, yet prognosis may be affected by treatment options and by the patient’s baseline characteristics. How PMF occurring as an SPC in CLL impacts patients’ prognoses has never been systematically investigated. On the other hand, however, it appears that in patients with MPNs, the development of SPC is not always associated with poor prognosis, and the exposure to cytoreductive drugs in this context has been identified as an independent predictor of survival [10]. In our second case, it is a matter of discussion whether the decision to not administer any PMF-directed therapy would have an effect on prognosis.

SPCs in CLL arise more frequently in different patient subsets; they are more common in males, in patients having undergone previous treatments, and in those with high-risk cytogenetics and the TP53 mutation [11]. Both of our patients presented with at least two of these risk factors, underlying the relevance of the clinical-biological setting for the development of CLL-associated MPNs.

Another intriguing finding in our cases is the temporal association between PMF development and the initiation of treatment with ibrutinib and venetoclax (6 months in case #1 and 8 months in case #2), hinting at a possible role of these two agents in favouring the emergence of the previously hidden PMF clone and its clinical phenotype. Interestingly, the opposite situation has been reported with JAK2 inhibitor therapy for PMF, which leads to the rapid occurrence of CLL [12]. A recent report has shown that ibrutinib therapy does not appear to reduce the risk of SPC, and does not significantly alter the types of malignancies associated with CLL [13]. No similar data regarding venetoclax are available on this matter. As the pathobiology of CLL and PMF may be more intertwined than previously thought, in our two patients, the suppression of the CLL clone might have indirectly favoured the PMF clone through various mechanisms. For instance, ibrutinib has the ability to alter the bone marrow microenvironment, contributing to its antitumor activity in CLL [14]. The effects of these alterations on another clone, however, are not known, and could theoretically be the opposite. Even more intriguing is the fact that both ibrutinib and venetoclax result in substantial degrees of immune dysregulation, by altering normal T, NK, B cell, and even monocyte/macrophage functions [15]. This could, in turn, lead to reduced immune surveillance on a PMF clone. 

## 3. Conclusions

While case reports offer an interesting chance to reflect on the mechanisms underlying the CLL/PMF association, all theories remain speculative, as no clear assumption can be made based on the two cases here reported, until more are included in the literature and new insights are gained into the connection between coexisting lymphoproliferative neoplasms and MPN. This may be achieved, perhaps, with future research aimed at specifically investigating pathogenetical mechanisms. More insights may also be gained as experience with the use of targeted agents grows, and their biological effects are further investigated. Given the large number of patients undergoing treatment with these agents, attention should be paid to the possibility of rare SPC arising in CLL, such as PMF, especially as the clinico-biological characteristics of this rare association are still largely unknown.

## Data Availability

The data presented in this study are available on request from the corresponding author.

## References

[1] Hallek M., Shanafelt T.D., Eichhorst B. (2018). Chronic lymphocytic leukaemia. Lancet.

[2] Hilal T., Gea-Banacloche J.C., Leis J.F. (2018). Chronic lymphocytic leukemia and infection risk in the era of targeted therapies: Linking mechanisms with infections. Blood Rev..

[3] Zent C.S., Kay N.E. (2010). Autoimmune complications in chronic lymphocytic leukaemia (CLL). Best Pract. Res. Clin. Haematol..

[4] Royle J.A., Baade P.D., Joske D., Girschik J., Fritschi L. (2011). Second cancer incidence and cancer mortality among chronic lymphocytic leukaemia patients: A population-based study. Br. J. Cancer.

[5] Holst J.M., Plesner T.L., Pedersen M.B., Frederiksen H., Møller M.B., Clausen M.R., Hansen M.C., Hamilton-Dutoit S.J., Nørgaard P., Johansen P. (2020). Myeloproliferative and lymphoproliferative malignancies occurring in the same patient: A nationwide discovery cohort. Haematologica.

[6] Marchetti M., Carobbio A., Capitoni E., Barbui T. (2018). Lymphoproliferative disorders in patients with chronic myeloproliferative neoplasms: A systematic review. Am. J. Hematol..

[7] Darawshy F., Ben-Yehuda A., Atlan K., Rund D. (2018). Chronic Lymphocytic Leukemia and Myelofibrosis. Case Rep. Hematol..

[8] Tadmor T., Shvidel L., Aviv A., Ruchlemer R., Bairey O., Yuklea M., Herishanu Y., Braester A., Levene N., Vernea F. (2013). Significance of bone marrow reticulin fibrosis in chronic lymphocytic leukemia at diagnosis: A study of 176 patients with prognostic implications. Cancer.

[9] Todisco G., Manshouri T., Verstovsek S., Masarova L., Pierce S.A., Keating M.J., Estrov Z. (2015). Chronic lymphocytic leukemia and myeloproliferative neoplasms concurrently diagnosed: Clinical and biological characteristics. Leuk. Lymph..

[10] Marchetti M., Ghirardi A., Masciulli A. (2020). Second cancers in MPN: Survival analysis from an international study. Am. J. Hematol..

[11] Visentin A., Imbergamo S., Gurrieri C., Frezzato F., Trimarco V., Martini V., Severin F., Raggi F., Scomazzon E., Facco M. (2017). Major infections, secondary cancers and autoimmune diseases occur in different clinical subsets of chronic lymphocytic leukaemia patients. Eur. J. Cancer.

[12] Sousos N., Buck G., Rodriguez-Meira A., Norfo R., Hamblin A., Pezzella F., Davies J., Hublitz P., Psaila B., Mead A.J. (2020). Rapid Emergence of Chronic Lymphocytic Leukemia During JAK2 Inhibitor Therapy in a Patient With Myelofibrosis. Hemasphere.

[13] Bond D.A., Huang Y., Fisher J.L., Ruppert A.S., Owen D.H., Bertino E.M., Rogers K.A., Bhat S.A., Grever M.R., Jaglowski S.M. (2020). Second cancer incidence in CLL patients receiving BTK inhibitors. Leukemia.

[14] Niemann C.U., Herman S.E., Maric I., Gomez-Rodriguez J., Biancotto A., Chang B.Y., Martyr S., Stetler-Stevenson M., Yuan C.M., Calvo K.R. (2016). Disruption of in vivo Chronic Lymphocytic Leukemia Tumor-Microenvironment Interactions by Ibrutinib—Findings from an Investigator-Initiated Phase II Study. Clin. Cancer Res..

[15] Svanberg R., Janum S., Patten P.E.M., Ramsay G.A., Niemann C.U. (2021). Targeting the tumor microenvironment in chronic lymphocytic leukemia. Haematologica.

